# Effect of grazing disturbance on floral display, pollen limitation and plant pollination efficiency in the desert steppe

**DOI:** 10.1186/s12870-022-03899-w

**Published:** 2022-11-04

**Authors:** Min Chen, Xue-Yong Zhao, Ping Yue, Xin-Xin Guo, Jing-Juan Qiao, Xiang-Yun Li

**Affiliations:** 1 Northwest Institute of Eco-Environment and Resources, CAS, Lanzhou, 730000 China; 2 Urat Desert-Grassland Research Station, Northwest Institute of Eco-Environment and Resources, Chinese Academy of Sciences, Lanzhou, China; 3 Key Laboratory of Stress Physiology and Ecology in Cold and Arid Regions, Lanzhou, 730000 Gansu Province China; 4 Naiman Desertification Research Station, Northwest Institute of Eco-Environment and Resources, Chinese Academy of Sciences, Lanzhou, China

**Keywords:** Grazing disturbance, Floral display, Pollen limitation, Pollinator activity, Seed production

## Abstract

**Background:**

Grazing disturbance usually affects floral display and pollination efficiency in the desert steppe, which may cause pollen limitation in insect-pollinated plants. Effective pollination is essential for the reproductive success of insect-pollinated plants and insufficient pollen transfer may result in pollen limitation. *Caragana microphylla* Lam is an arid region shrub with ecological importance. Few studies have been conducted on how grazing disturbance influences pollen limitation and pollination efficiency of *C. microphylla*. Here, we quantify the effect of different grazing intensities on floral display, pollinator visitation frequency and seed production in the Urat desert steppe.

**Results:**

In *C. microphylla*, supplemental hand pollination increased the seed set, and pollen limitation was the predominant limiting factor. As the heavy grazing significantly reduced the seed set in plants that underwent open-pollination, but there was no significant difference in the seed set between plants in the control plots and plants in the moderate grazing plots. Furthermore, there was a higher pollinator visitation frequency in plants in the control plots than in plants in the heavy grazing plots.

**Conclusions:**

We found that pollinator visitation frequency was significantly associated with the number of open flowers. Our findings also demonstrated that seed production is associated with pollinator visitation frequency, as indicated by increased seed production in flowers with higher pollinator visitation frequency. Therefore, this study provides insight into the effect of different grazing intensities on floral display that are important for influencing pollinator visitation frequency and pollination efficiency in desert steppes.

**Supplementary Information:**

The online version contains supplementary material available at 10.1186/s12870-022-03899-w.

## Background

Grassland desertification characterized by vegetation degradation is predominantly caused by continuous heavy grazing [1]. Long-term grazing can be related to decreased vegetation cover, plant height and number of open flowers, thus influencing floral display and the reproductive success of plants in desert steppes [2–4]. Heavy grazing alters plant and insect communities, and the plant–pollinator relationship is sensibility to the anthropogenic effects of habitat change [5]. Grazing influences individual plant growth and population dynamics and can change vegetation characteristics in desert steppe ecosystems, resulting in different grazing intensities that may exhibit variation in plant floral display [6]. The variation in floral display may reflect environmental factors such as pollen limitation and resource limitation [7]. Many species employ rewards and more open flowers as methods for attracting pollinators, and pollinators are usually less attracted to plants with low-density flowers, which generally receive less pollen than plants with high-density flowers [8]. The higher livestock grazing intensity can even negatively affect pollinator species richness and abundance [5]. In that regard, understanding the mechanisms through which grazing may affect pollinator assemblage is critical for informed management decisions and insect-pollinated conservation planning.

Pollen limitation occurs when plants receive insufficient pollen, reducing the reproductive success of the plant [9]. The global expansion of livestock grazing, particularly in desert areas, is considered a major threat to pollination services [5]. The plant–pollinator relationship is a good barometer of interaction biodiversity under anthropogenic effects due to its sensibility to habitat change [10]. Having numerous open flowers does not overcome pollen limitation due to low pollinator visits, and the inefficiency of pollinators is the dominant cause of insufficient pollen transfer [9]. Floral traits and display may increase resource acquisition, ultimately influencing pollination efficiency [11, 12]. Furthermore, livestock-associated reductions in floral resources may be insufficient to maintain pollinator populations, resulting in their migration to more resource-rich locations [10].

Floral display of a plant species may influence pollen limitation intensity directly or indirectly [13–15]. Floral display may function not only to facilitate pollinator visits but also to restrict pollinator efficiency [9, 16]. In the flowering period, plants with more open flowers usually provide a stronger reward signal (pollen and nectar) and attract more visits from pollinators [7, 17]. The quality of pollen that reaches the stigma, the behavior of pollinators, and the efficiency of pollen delivery are major biotic factors affecting reproductive success [18]. In addition, Karron and Mitchell (2012) pointed out that open flower number is one of the most important quantitative display that directly affects offspring quantity and quality [19]. Most of the plants that tend to be visited by more effective pollinators in response to pollinator selection have floral display with adaptive pathways [20]. Floral specialization is driven by interactions with pollinators, but more attention should be given to floral display [21].

*Caragana microphylla* is an economically important species and has great potential as a forage grass for sheep. This species is a drought-resistant and sand-fixation plant, allowing it to contribute to the stability of the ecosystem of the desert steppes [4]. Long grazing history had significantly influenced the characteristics of reproduction of *Caragana microphylla* under different grazing intensities [22]. Many studies have demonstrated above plant biomass changes, but no study has examined the effects of heavy grazing on reproductive success [6, 23]. The overall objective of this study was to reveal how different grazing intensities and pollen limitation affect the reproductive success of *C. microphylla*. This study aimed to: 1) determine whether *C. microphylla* experiences pollen limitation and possible differences in seed set under different grazing treatments; 2) evaluate the correlation between open flower number and pollinator visitation frequency; and 3) examine how pollinator visitation affects seed production based on different grazing intensities.

## Results

### Different grazing intensities and rainfall affect vegetation

**Fig. 1:**
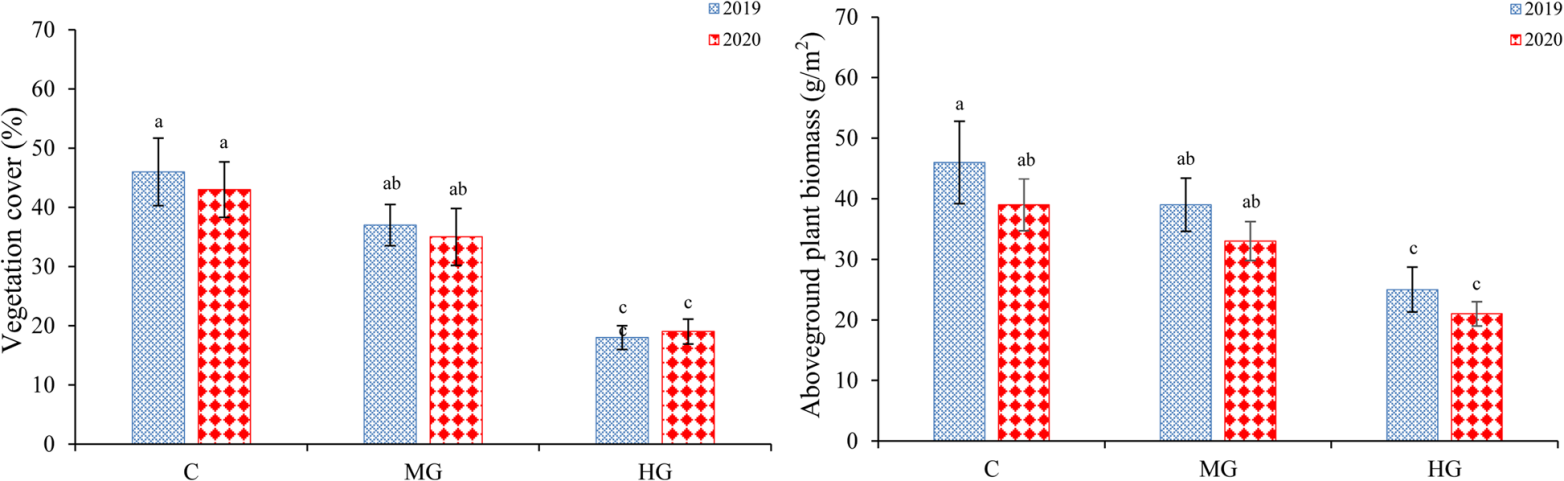
Effects of different grazing intensities on vegetation. Vegetation cover (**A**) and aboveground plant biomass (**B**) of *C. microphylla*. C, control; MG, moderate grazing; and HG, heavy grazing

In the control, the vegetation cover (VC) was 43.6 ± 3.7 (Mean ± SD) and aboveground plant biomass (AGB) was 39.0 ± 3.1in 2020 (Fig. 1A, B.). Our results indicated that the control had a higher mean VH and AGB than those in the grazing plots (*df* = 1, *P* < 0.05). In addition, the VC and AGB of control was not significantly different between 2019 and 2020 (*df* = 1, *P* > 0.05). The growing period of *C. microphylla* typically occurs from May until mid-June, and we also recorded the total rainfall per ten days in each year (Supplementary; Figure S1). Our results indicated that the total rainfall per ten days in 2019 was higher than in 2020 but their total rainfall were very small.

### Floral display

Our results demonstrated that the mean number of open flowers was 10.2 ± 2.1 (Mean ± SD) and the mean corolla sizes was 162.3 ± 32.2 (Table 1). Our results also indicated that corolla size was not significantly different among the different grazing plots. We observed that the mean number of open flowers in the control and heavy grazing plots was 10.6 ± 2.1 and 8.3 ± 1.2, respectively. The number of open flowers in the control was significantly higher than that in the heavy grazing treatment (*P* < 0.001). In addition, the number of open flowers was not significantly different between the control and the moderate grazing intensity (*P* > 0.05).

**Table 1:** Floral traits (Mean ± SD) of *C. microphylla* between the control and the different grazing plots

Traits	Control	MG	*P*	Control	HG	*P*
Number of open flowers	10.6 ± 2.1	9.8 ± 1.8	*P* < 0.01	10.6 ± 2.1	8.3 ± 1.2	*P* > 0.05
Corolla size (mm^2^)	173.5 ± 23.8	161.2 ± 17.6	*P* < 0.01	173.5 ± 23.8	143.6 ± 12.9	*P* < 0.05

### Pollen limitation

**Fig. 2:**
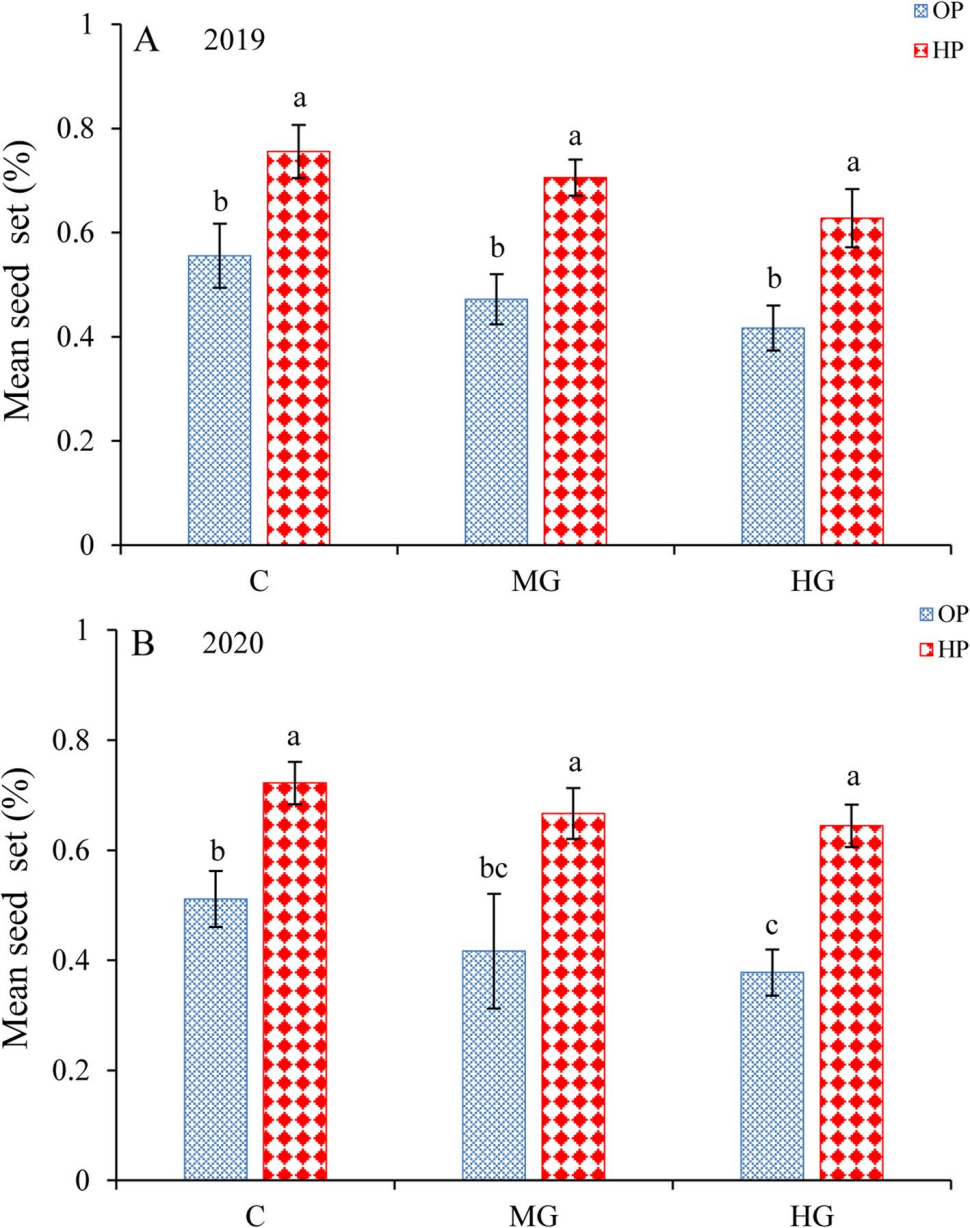
The mean seed set of *C. microphylla* under different grazing intensities (C, MG and HG), treatments (OP and HP) and years (2019 and 2020). OP, open pollination treatment; HP, hand pollination treatment. Different letters indicate a significant difference at the 0.05 level

For each pollination treatment, the mean seed set from 2019 to 2020 is shown in Fig. 2A and B. In the control, the mean seed set of the open-pollinated treatment was 51.1 ± 5.0% (Mean ± SD), while the mean seed set of the hand-pollinated treatment was 72.2 ± 3.9% in 2020. For heavy grazing, there was a significant difference between the open-pollinated and pollen addition treatments at 37.8 ± 4.2% (open-pollinated) and 64.4 ± 3.9% (hand-pollinated; treatment effect, likelihood ratio χ^2^ = 50.174, *df* = 1, *P* < 0.001; Table 2) in 2020. In addition, the hand-pollinated samples had a higher mean seed set than the open-pollinated samples. Our results indicated that *C. microphylla* exhibited severe pollen limitation, and the pollen limitation index for the heavy grazing plots was 0.41 in 2020. Furthermore, as the grazing intensity increased, the pollen limitation index increased.

**Table 2:** Impact of different grazing intensities (C, MG and HG), treatments (OP and HP) and years (2019 and 2020) on the seed set of *C. microphylla*. OP, open pollination treatment; HP, hand pollination treatment

	Seed set		
	Likelihood ratio χ^2^	*df*	*P*
Different grazing intensities	16.877	2	*P* < 0.001
Treatments	50.174	1	*P* < 0.001
Years	2.486	1	0.115

For open-pollinated samples, the mean seed set of the control plots (51.1 ± 5.0%) was significantly higher than the mean seed set of the heavy grazing plots in 2020 (37.8 ± 4.2%; different grazing intensities, likelihood ratioχ^2^ = 16.877, *df* = 2, *P* < 0.001; Table 2). Our results indicated that the heavy grazing plots showed a significantly reduced seed set, but the seed set was not significantly different between the control and moderate grazing plots (*P* > 0.05).

### Pollinator visitation frequency and activity

**Fig. 3:**
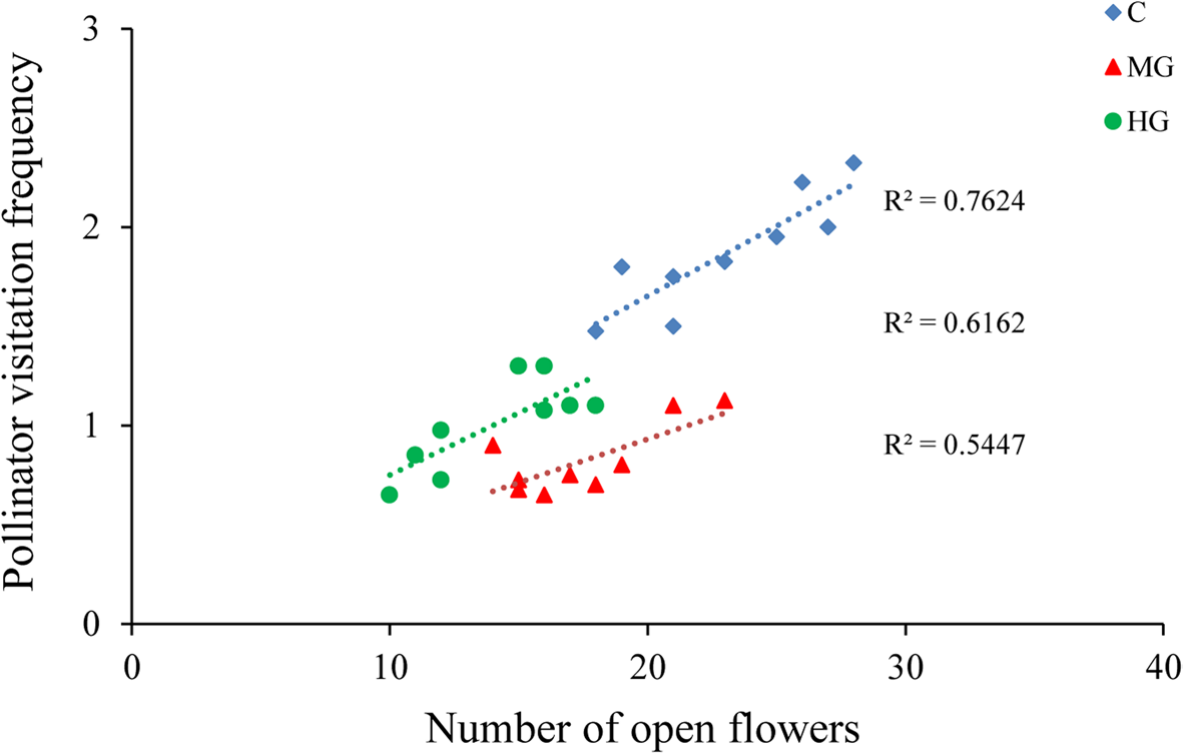
Relationship between the pollinator visitation frequency and the number of open flowers in different grazing intensities

In the flowering period, our results indicated that *Apis mellifera* accounted for 86.0% of the 267 pollinators observed in the control plots. *A. mellifera* had a significantly higher number of visits than other pollinators (*P* < 0.05), and this species was the dominant pollinator. In blooming period, the flower opening occurred between 08:00 and 18:00. *A. mellifera* were the first visitors in the morning, and they pushed the tepals out to enter these flowers. The most frequent activity of *A. mellifera* coincided with this time (Supplementary; Figure S2). In addition, there is a tripping mechanism in the flowers of *C. microphylla*, and pollinator visitation activity acts as a tripping agent. Other occasional visitors included *Megachile* (Chalicodoma) *desertorum* Morawitz and *Episyrphus balteatus* (Supplementary; Table), these species only play an assistant role in pollination success due to their infrequent visitation and because they rarely touch the stigma or anthers. Pollinator visitation frequency was significantly associated with the number of open flowers in the studied plots (Fig. 3). In addition, there were significant differences in the number of pollinator visits between the control and the heavy grazing plots (*P* < 0.05; Fig. 3).

### Pollinator visitation affects seed production

**Fig. 4:**
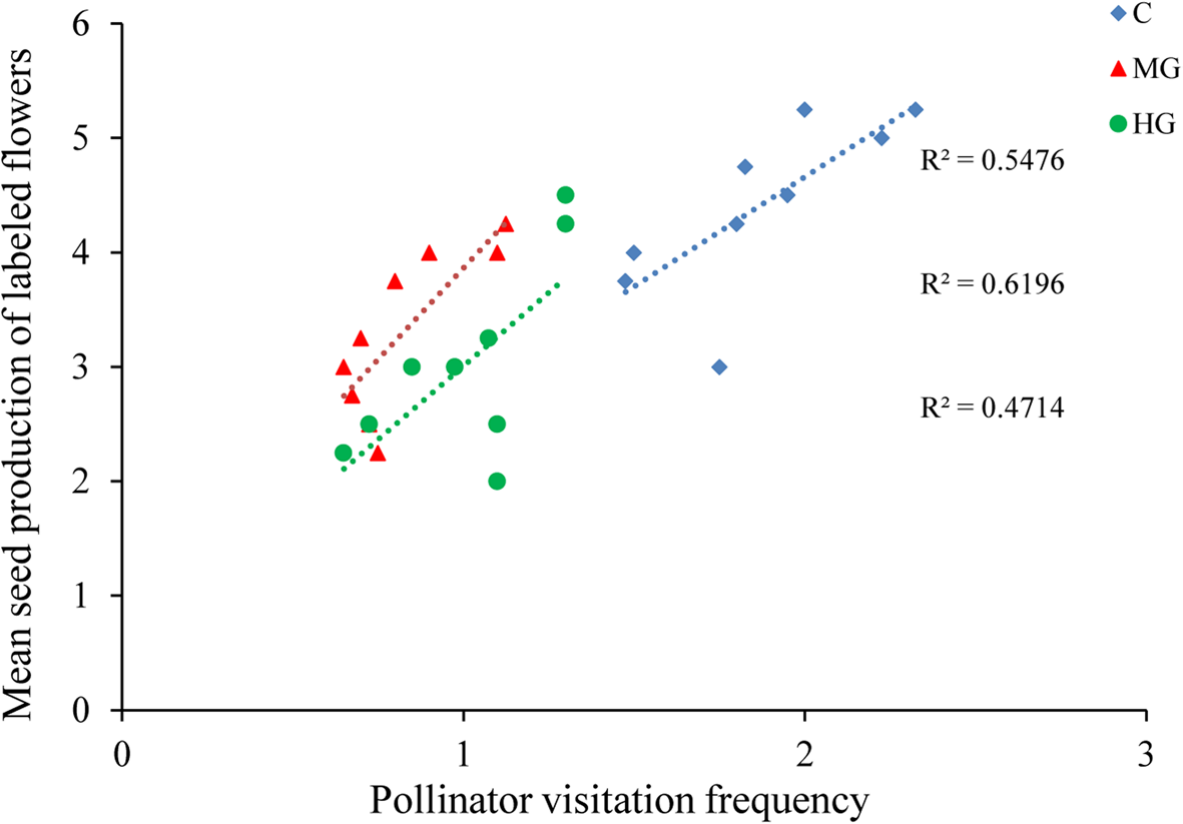
Relationship between the mean seed production of labeled flowers and the pollinator visitation frequency in different grazing intensities

In the marked flowers, the mean pollinator visitation frequency (VF) was 1.2, with most plants visited at least once by effective pollinators, and labeled flowers produced a mean number of seeds of 3.6 (NS). These results showed that the seed production of marked flowers was significantly correlated with pollinator visitation frequency in the control plots (seed production among visited flowers: *r* = 0.56, *P* < 0.01; Fig. 4).

## Discussion

### Floral display and pollinators under grazing disturbance

Many studies have explored the impacts of grazing disturbance on plant reproduction and diversity and assessing how different grazing intensities affect floral display and pollinator activity is key to understanding the pollination ecology of desert steppes [4, 24]. Tadey (2015) pointed out that the high grazing intensity can cause an indirect negative effect by reducing the floral resources because the heavy grazing can influence the bee-plant interaction networks [10]. The function of floral display is not only to improve pollination success by the dominant pollinator but also to restrict visitation by other potential pollinators, and these traits may reduce the transfer pollen efficiency of potential pollinators [7, 25, 26]. Heavy grazing and extreme environmental conditions can reduce pollinator visits, as reduced floral rewards cannot satisfy the pollination requirements of pollinators [21]. In the present study, we found that pollinator visits acted as a tripping agent in *C. microphylla*. In the flowering period of *C. microphylla*, pollen release and pollinator visitation activity were highly consistent, indicating an adaptation mechanism for increasing pollination efficiency in desert steppes. The related research pointed out that heavy grazing significantly reduced the number of open flowers and height of *C. microphylla*, but moderate grazing hadn’t an obvious negative effect on the number of open flowers [27]. Our findings provide further support for these points, we suggested that appropriate stocking rate should be moderate grazing (0.5 sheep per ha) in the Urat desert steppe. We also found the total rainfall per ten days in 2019 and 2020 were very small and they have little effect on plants growing. Furthermore, the VC and AGB of control was not significantly different between 2019 and 2020.

Floral display attract pollinators, and an association between flower resources and pollinator visitation frequency might exist [28]. Pollinator visitation frequency seems to be a good predictor of pollination efficiency, and areas with greater pollinator visitation frequencies have higher pollination efficiency [29]. Reduced pollinator activity could threaten plant pollination success when pollinators are inadequate [30]. Heavy grazing alters plant community structure, vegetation cover, and changes in soil moisture in grasslands [5, 31]. Furthermore, the behavior of grazing livestock mostly have detrimental effects on the visiting activities of pollinator [32]. Our results also demonstrated that heavy grazing disturbance reduced vegetation cover and the number of open flowers, and floral display affected the pollinator visitation frequency and the activity of pollinators. As the grazing intensity decreased, we found that *C. microphylla* exhibited a higher pollinator visitation frequency in the control plots than in the grazing disturbance plots. This study illustrates that the impacts of grazing intensity on floral display are important for influencing pollinator visitation frequency.

### Pollen limitation and pollinators

Pollen limitation commonly occurs when pollinators are rare or when the transfer of pollen by pollinators is ineffective [7, 26]. Many flowering plants are more vulnerable to pollen limitation due to their reliance on pollinators for pollination success [29]. Most insect pollinated plants show evidence of inadequate pollen receipt when pollinators are scarce, or when plants are self-pollinated [25]. Plants can evolve mechanisms of reproductive assurance, and floral display may evolve in response to pollen limitation [4]. Glaettli and Barrett (2008) demonstrated that the number of open flowers was positively associated with pollinator visitation frequency, resulting in effective pollination efficiency [33]. Furthermore, plants with greater numbers of open flowers provide a strong signal of greater “rewards” and hence may attract more pollinator visits, ultimately resulting in the production of more seeds [17]. Insect pollinated plants often experience pollen limitation due to unreliable pollinator services, and plant reproduction may be limited by inadequate pollen receipt or resource availability [15]. Our findings demonstrated that *C. microphylla* experienced severe pollen limitation in heavily grazed plots and that pollen addition was the greatest limiting factor for seed set.

Nectar and pollen are the main targets of pollinators, thus, floral display can attract pollinators to particular flowers [7]. The quantity of open flowers is an important floral display that can directly influence the reproductive success of flowering plants [17]. Many plant species relying on less effective pollinators may experience serious declines in pollination success if a harsh environment and human interference affect pollinator activity [25]. To improve predictions of plant pollination success, it is important to understand the relationship between pollen limitation and pollinators. In this study, our results suggested that pollinator visitation frequency was significantly positively associated with the number of open flowers. Furthermore, we found that grazing disturbance influenced the number of open flowers and pollinator visitation frequency and seed production was positively associated with pollinator visitation frequency.

### Plant pollination efficiency under different grazing intensities

Grazing disturbance tends to decrease vegetation cover and plant height and changes to floral display can also reflect plant adaptation to grazing disturbance [4, 23, 34] Changes in pollinator richness with the intensity of livestock grazing was mediated by the effect of grazing on the floral resources [35]. Many studies have indicated that floral display may influence pollinator visitation frequency and the efficiency of resource transfer [16, 21]. The transfer of pollen is an important biotic factor that can affect seed production in animal-pollinated plants [29]. Pollinator activity is an effective pollination model, and reduced pollinator visitation may lead to a decrease in the quantity and quality of cross-pollen transfer, resulting in a reduction in seed production [36]. In this study, we compared results from plots subjected to different grazing intensities in a desert steppe. The decreased pollinator visitation frequency observed in plants from heavy grazing plots was associated with a lower probability of seeding in *C. microphylla*. In addition, pollinator visitation frequency may explain why the control plots exhibited a higher rate of seed production than plots subjected to grazing. We found that plants in the grazing plots experienced fewer pollinator visits and suffered stronger pollen limitation, resulting in a lower seed set than that in plants in the control plot.

Effective pollinators spent more time in open flowers and visited regions with greater resources [36, 37]. A similar study suggested that the number of open flowers was positively correlated with fruit and seed production [38]. In addition, this species (*C. microphylla*) has developed adaptive strategies for its heavy grazing because of the effects of grazing on dominant plant population in the desert steppe [39]. Clonal reproduction plays an important role in reproduction success of *C. microphylla* [40]. In the present study, we also found that plants with more open flowers exhibited an advantage with respect to plant pollination efficiency. Moderate grazing has been shown to sustain floral display, whereas heavy grazing can result in a significant decline in floral display and plant reproduction success. Higher livestock grazing intensity was associated with a loss of open flowers in plants, affecting a decline in pollinator visitation frequency [41]. Vulliamy et al., (2006) pointed out that pollinators feed mostly on nectar and pollen, the availability of floral resources is a major driver of pollinator activity [42]. Furthermore, this finding was supported by our results indicating that heavy grazing significantly reduced the seed set in plants receiving the open-pollinated treatment, but there was no significant difference in seed set between the control and moderate grazing plots. Understanding the mechanisms through which different grazing intensities may affect pollinator visiting is critical for informed management decisions and plant-pollinator conservation planning [35]. Our findings are important for understanding the effect of different grazing intensities on floral display, pollinator visits and the pollination success of *C. microphylla*.

## Conclusions

We conclude that heavy grazing weakens plant pollination efficiency through the decrease of pollinator visitation frequency. Heavy grazing lead to a substantial decline in the number of open flowers. Furthermore, the number of open flowers and pollinator visitation frequency were positively correlated. Therefore, we found more seed production in flowers with higher pollinator visitation. Our study strongly speaks for setting upper limits to livestock grazing in the desert steppe of in Inner Mongolia as an insect-pollinated plants conservation strategy. Controlling grazing intensity via the use of fencing may be an effective way to increase pollination efficiency and promote rangeland sustainability in desert steppe.

## Materials and methods

### Plant species

In *C. microphylla*, the flowering period occurs from May until June, and fruiting most takes place in August. In addition, *C. microphylla* is only a part self-compatible and insect pollination plays an important role in the breeding system [22]. We have permission to collect *C. microphylla*, MC undertook the formal identification of *C. microphylla* used in our study. Furthermore, we confirm that a voucher specimen of *C. microphylla* has been deposited in a publicly available herbarium.

### Study area

The study was carried out at the Urat desert steppe in the provinces of western Inner Mongolia, China (106°59'-107°05'E, 41°06'-41°25'N). The average annual precipitation from 1971 to 2011 was approximately 140 mm, and the greatest period of rainfall is from May to September [23]. The study area is the shrub-dominated community. In addition, the dominant plant species is *Caragana microphylla* Lam, and there are a small amount of *Reaumuria songarica* (Pall.) Maxim. The dominant species is the managed populations which has the same age.

### Grazing experimental design

**Fig. 5:**
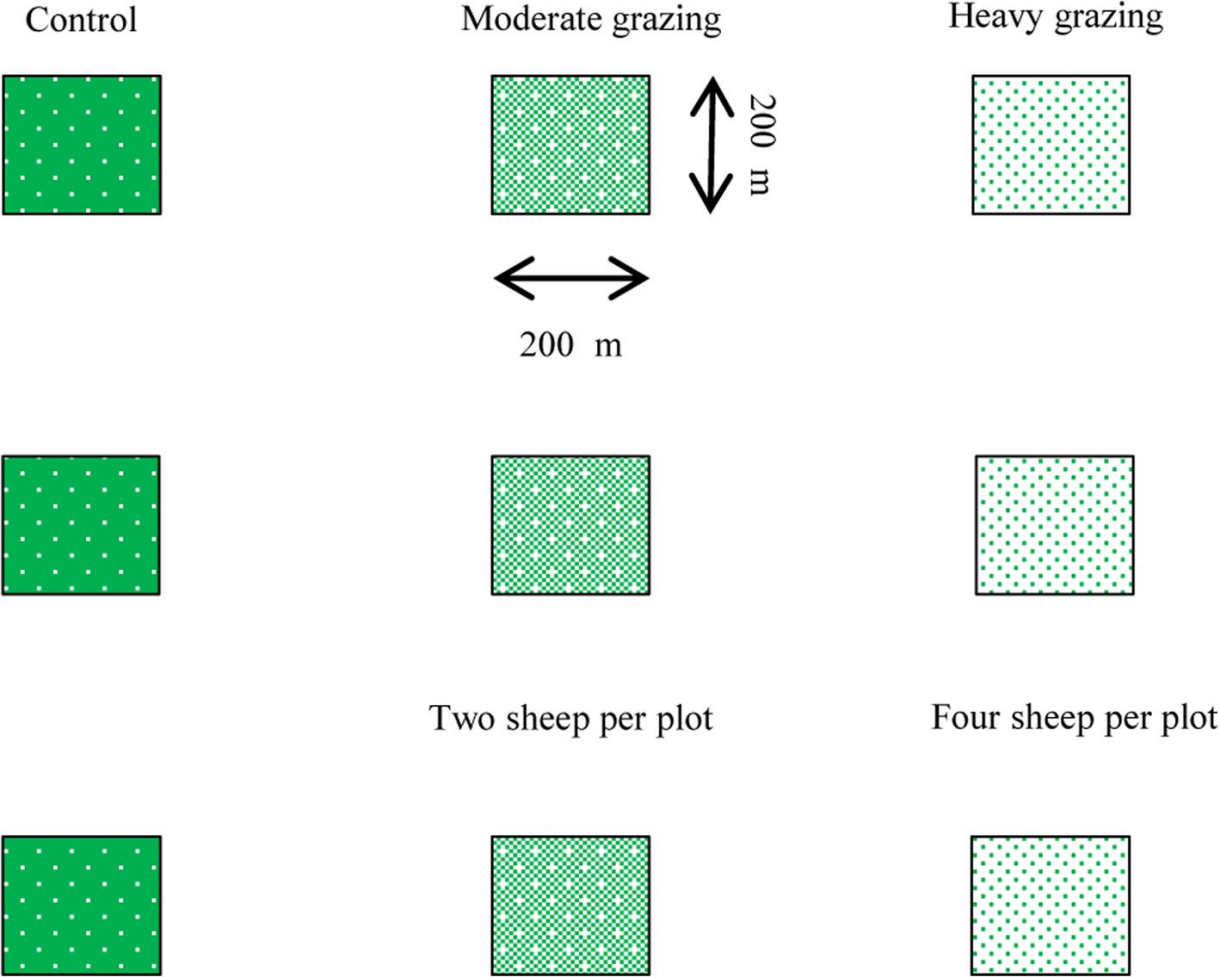
Experimental layout for the grazing experiments, control, moderate grazing and heavy grazing plots, from 2019 to 2020. There are nine plots (200 m × 200 m), moderate grazing and heavy grazing plots were used fence to separate sheep from other plots

Grazing experiments have been carried out in the study area since 2013. This study was performed from May 2019 to October 2020. We also calculated the monthly rainfall in 2019 and 2020 in the study area. According to the grazing capacity of desert steppe in Inner Mongolia, we selected three grazing intensities: control plot (no grazing), moderate grazing plot (two sheep per plot) and heavy grazing plot (four sheep per plot) [23]. The plots were all approximately four ha and the study plots were protected from other human impacts. There were three replicates (plots) for each grazing intensity and the experimental layout comprised a total of nine grazing plots (Fig. 5). The sheep freely grazed in the enclosed fences in the day time and they were raised in the sheep pens under the same conditions at night. We selected two-year old sheep with similar weight and healthy for the grazing experiments. In addition, these selected sheep were replaced by the new two-year old sheep every three years [23]. In each plot, we set up five 2 m × 2 m quadrats at the center and the four corners respectively. Vegetation measurements were conducted in August, when the standing aboveground biomass reached the peak. For each quadrat, we used the projection method to measure the vegetation cover (VC) and then harvested aboveground biomass (AGB) of each species. We have dried samples in an oven using 65℃ for 48 h to constant mass and weighed.

### Measured floral display

To assess floral display under different grazing intensities (control, moderate grazing and heavy grazing), we randomly marked nine similar plants in each plot. The single flower period lasted for five days. In blooming period, we selected three inflorescences in each marked plant and counted the number of open flowers during the five days. In the selected flowers, the corolla size (width and height) was recorded with digital calipers [12].

### Estimation of pollen limitation

To determine whether this species experiences pollen limitation leading to reduced seed set, nine marked plants were assigned to open pollination and another nine plants were assigned to receive supplemental hand pollination from 2019 to 2020 [9, 43]. In each marked plant, we selected twelve open flowers (three inflorescences per plant, four flowers per inflorescence); this was done for all three grazing intensities. For the supplemental hand pollination, the additional pollen was collected from random unmarked individuals at a minimum distance of 10 m away. In September, we recorded and counted the seed set of marked flowers for the different grazing intensities. We evaluated the pollen limitation index: PL index = 1—(S_OP_/S_HP_), where S_OP_ is the seed set from flowers receiving open-pollinated treatment and S_HP_ is the seed set from flowers receiving the hand-pollinated treatment [44].

### Pollinator visitation frequency and pollinator activity

To evaluate the relationships between the number of open flowers and pollinator visitation frequency for different grazing intensities, we selected six flowering plants for observation in each plot. In each selected plant, we randomly marked 12 open flowers (three inflorescences per plant, four flowers per inflorescence and removed other open flowers). In the flowering period, we labeled a total of 72 open flowers in each plot. We performed 10 h focal observations from 08:00 to 18:00 in each day. HD camera was used to the duration of each pollinator visit, the time until pollinators visited the plot, and the number of plants and number of flowers visited per foraging bout. Six surveyors used 70 h (10 h per day) to record pollinator activity because each observation period was one week. We used the pollinator visitation frequency [45]:

$$\text{Visitation frequency }=\frac{\text{N}_\text{V}\,}{\text{N}_{\text{F}} * \text{T}},$$

where N_V_ is No. pollinator visits, N_F_ is No. open flowers and T is the observation time of pollinators (hour).

### Pollinator visitation affects seed production

To examine the effect of pollinator visitation on seed production, six flowering plants were randomly marked under natural conditions in each grazing plot. We marked four flowers in each selected inflorescence (three inflorescences marked per plant). We used an HD camera to record the pollinators visiting marked flowers and used insect nets to capture pollinators. In addition, we used a fuchsin-stained jelly to rub pollen from the pollinator body, and we later identified the pollen from the pollinator by using a stereomicroscope in the laboratory. After the observation time, the pollinator visited flowers were covered with bags to eliminate interference from other pollinator visits and wind pollination. We used seed production as an important indicator of pollination success and recorded pollinator visitation frequency and seed production from May until September. We examined the effect of pollinator visitation on seed production by assessing the relationship between the mean seed production of marked flowers (NS) and the frequency of pollinator-visited flowers (VF).

### Data analyses

A generalized linear model (GLM) with a gamma distribution and logit link function was used to assess the effects of pollination treatments (open-pollinated and hand-pollinated), different grazing intensities (control, moderate and heavy grazing), and years (from 2019 to 2020) on seed production. We considered tag number as a random factor within the different grazing intensities. We considered pollination treatments, grazing intensities and years as fixed factors, and seed set was the dependent variable in the model. We used a likelihood ratio test to determine the variations in different grazing intensities and applied Tukey’s method to adjust for multiple comparisons.

GLM was used to determine whether grazing intensity and year affected vegetation cover and aboveground plant biomass. We used one-way ANOVA to compare the number of open flowers and pollinators. In addition, we used one-way ANOVA to test multi-group comparisons of the means and post hoc contrasts were performed using the S–N–K test.

We used regression to evaluate the relationship between the mean seed production of marked flowers and pollinator visitation frequency, with pollinator visitation frequency as the independent variable and mean seed production among labeled flowers as the dependent variable. We used SPSS 22.0 to perform these analyses.

## Supplementary Information

**Additional file 1: Supplementary Figure S1.** The total rainfall per ten days in 2019 and 2020.

**Additional file 2: Supplementary Figure S2.** The frequency of dominant pollinator visits to *C. microphylla* flowers.

**Additional file 3: Supplementary Table S1.** The list of dominant and occasional pollinators in *C. microphylla.*

## Data Availability

All of the data on which conclusions rely in this study are included in this published article.
