# Supplementary figures and images for: Effect of grazing disturbance on floral display, pollen limitation and plant pollination efficiency in the desert steppe
Source: BMC Plant Biol. 2022 Nov 4;22:514. doi: 10.1186/s12870-022-03899-w (PMC9635133; doi:10.1186/s12870-022-03899-w)

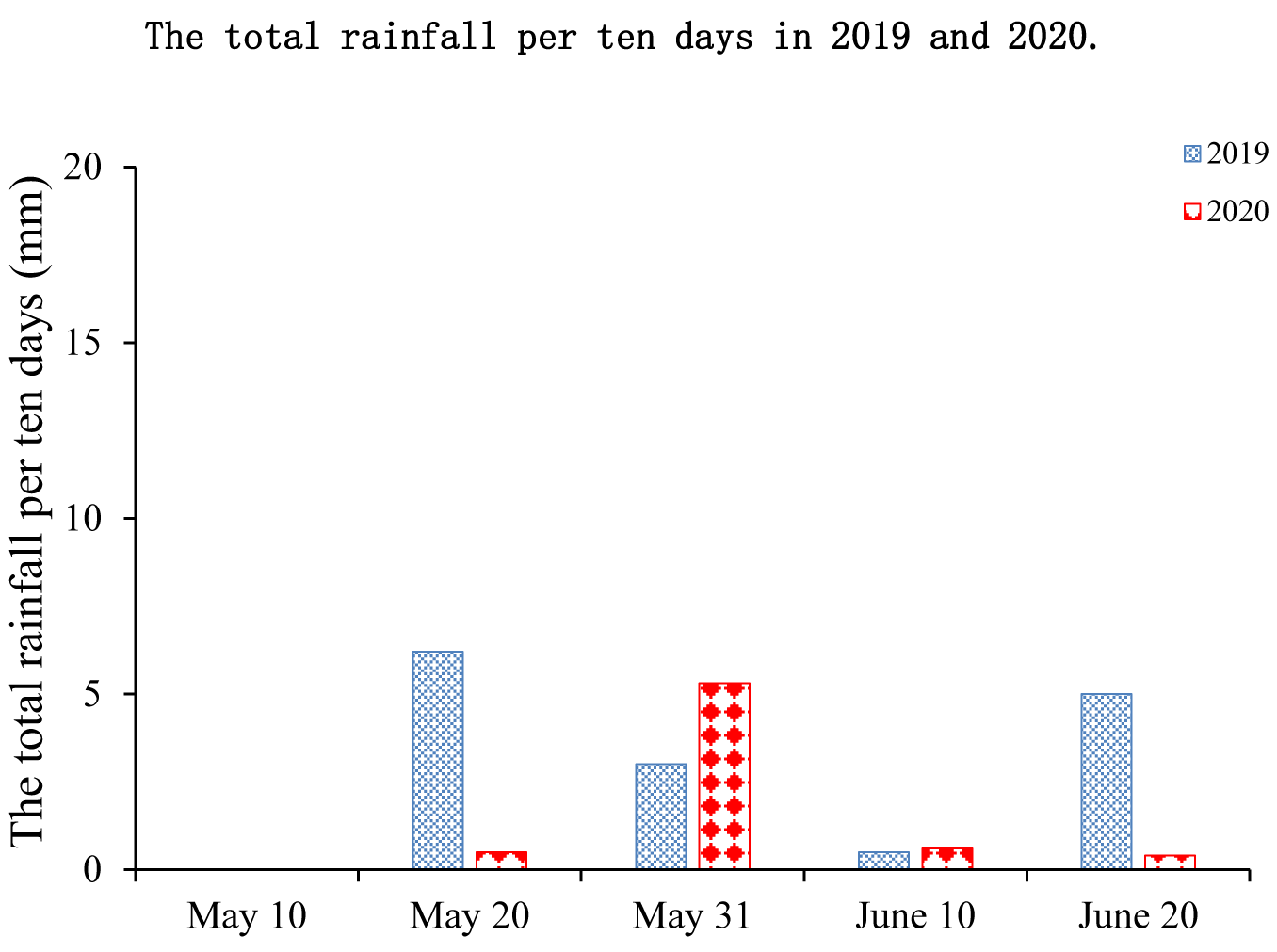

Supplement: Supplementary file 1 — Additional file 1: Supplementary Figure S1. The total rainfall per ten days in 2019 and 2020. [file 12870_2022_3899_MOESM1_ESM.tif]

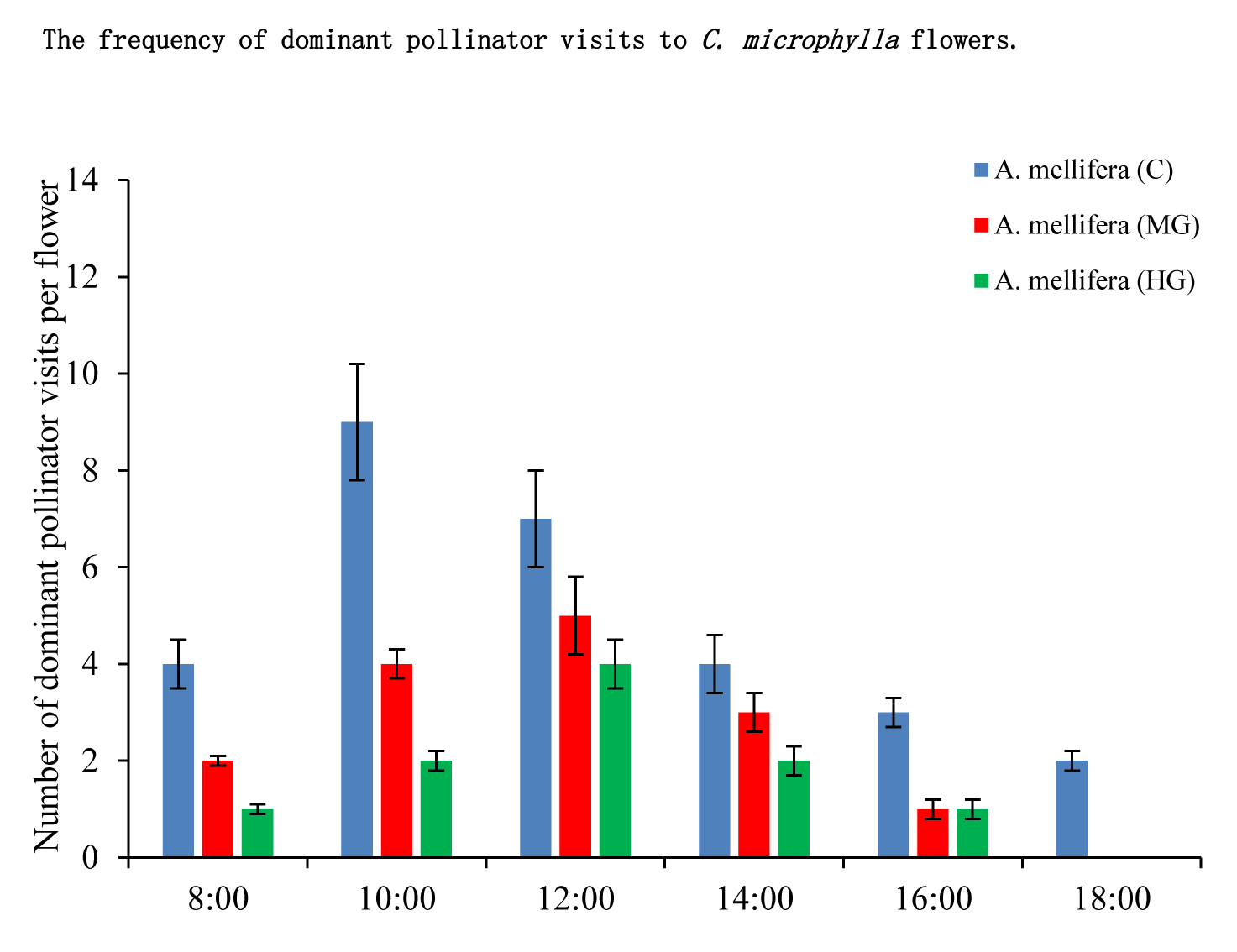

Supplement: Supplementary file 2 — Additional file 2: Supplementary Figure S2. The frequency of dominant pollinator visits to C. microphylla flowers. [file 12870_2022_3899_MOESM2_ESM.tif]
