# Supplementary material for: Effect of grazing disturbance on floral display, pollen limitation and plant pollination efficiency in the desert steppe
Source: BMC Plant Biol. 2022 Nov 4;22:514. doi: 10.1186/s12870-022-03899-w (PMC9635133; doi:10.1186/s12870-022-03899-w)
Supplement: Supplementary file 3 — Additional file 3: Supplementary Table S1. The list of dominant and occasional pollinators in C. microphylla. [file 12870_2022_3899_MOESM3_ESM.docx]

**The list of dominant and occasional pollinators in *C. microphylla*.**

|  | Order | Family | Genus |
| --- | --- | --- | --- |
| *Apis mellifera* | Hymenoptera | Apidae | Apis |
| *Megachile* (Chalicodoma) *desertorum* Morawitz | Hymenoptera | Megachilidae | Megachile |
| *Episyrphus balteatus* | Diptera | Syrphidae | Episyrphus |
